# Debating Euthanasia and Physician-Assisted Death in People with Psychiatric Disorders

**DOI:** 10.1007/s11920-022-01339-y

**Published:** 2022-06-09

**Authors:** Luigi Grassi, Federica Folesani, Marco Marella, Elisa Tiberto, Michelle B. Riba, Lisa Bortolotti, Tommaso Toffanin, Laura Palagini, Martino Belvederi Murri, Bruno Biancosino, Maria Ferrara, Rosangela Caruso

**Affiliations:** 1 Institute of Psychiatry, Department of Neuroscience and Rehabilitation, University of Ferrara, Via Fossato di Mortara 64a, 44121 Ferrara, Italy; 2 University Hospital Psychiatry Unit, Integrated Department of Mental Health and Pathological Addiction, S. Anna University Hospital and Local Health Trust, Ferrara, Italy; 3 Department of Psychiatry, University of Michigan, University of Michigan Rogel Cancer Center, Ann Arbor, MI USA; 4 Department of Philosophy in the School of Philosophy, Theology, and Religion and Institute for Mental Health in the School of Psychology, University of Birmingham, Birmingham, UK

**Keywords:** Euthanasia, Medical assistance in dying, Moral philosophy, Moral distress, Schizophrenia, Mental illness, Major depression, Psychosis, Personality disorders

## Abstract

**Purpose of Review:**

Over the last 30 years, medical assistance in dying (MAiD) including euthanasia (EU) and physician-assisted death (or suicide, PAS) has become the center of a large debate, particularly when these practices have involved people with psychiatric illness, including resistant depression, schizophrenia, personality, or other severe psychiatric disorders. We performed a review utilizing several databases, and by including the most relevant studies in full journal articles investigating the problem of MAiD in patients with psychiatric disorders but not in physical terminal conditions (non-terminal, MAiD-NT).

**Recent Findings:**

Literature has shown that a small percentage of people with psychiatric disorders died by MAiD-NT in comparison with patients with somatic diseases in terminal clinical conditions (e.g., cancer, AIDS). However, the problem in the field is complex and not solved yet as confirmed by the fact that only a few countries (e.g., the Netherlands, Belgium, Luxemburg) have legalized MAiD-NT for patients with psychiatric disorders, while most have maintained the practices accessible only to people with somatic disease in a terminal phase. Also, how to make objective the criterion of irremediability of a mental disorder; how to balance suicide prevention with assisted suicide; how to avoid the risk of progressively including in requests for MAiD-NT vulnerable segments of the population, such as minors, elderly, or people with dementia, in a productive-oriented society, are some of the critical points to be discussed.

**Summary:**

The application of MAiD-NT in people with psychiatric disorders should be further explored to prevent end-of-life rights from contradicting the principles of recovery-oriented care.

**Supplementary Information:**

The online version contains supplementary material available at 10.1007/s11920-022-01339-y.

## Introduction

Over the last 30 years, death with dignity has been a part of a large thematic discussion regarding hastening the patient’s death to put an end to their suffering, when no cure can be found such as patients in terminal stages of a somatic disease (e.g., advanced cancer, AIDS, amyotrophic lateral sclerosis). This topic, often subsumed under the rubric of medical assistance in dying (MAiD), namely, euthanasia (EU) and physician-assisted death (or physician-assisted suicide, PAS), has involved several areas of medicine, especially palliative medicine (Table 1 for definitions).

**Table 1:** Definitions about medical actions causing the patient’s death

1. *Active euthanasia:* intentionally causing the death of a patient by administering a lethal medication
a. *Voluntary:* causing the death of a competent patient at that person’s voluntary and explicit request
b. *Involuntary:* causing the death of a patient without his or her consent when he or she is competent to give consent but did not explicitly request it
c. *Non-voluntary:* causing the death of a patient who has irrevocably lost – or never had – the capacity for competence, so without any consent from him/her nor any explicit request
2. *Physician-assisted death* (or *physician aid in dying* or *physician-assisted suicide*): intentionally helping a person to end his/her life by administering or providing drugs for self-administration, at that competent person’s voluntary and explicit request
3. *Passive euthanasia* (an incorrect concept) to indicate *withdrawing/withholding treatment*: withdrawing or withholding life sustaining supports (i.e., stopping or withdrawing life-support measures, or refraining from starting them, such as *do not resuscitate* order) which will be followed by the death of the person, as usually he/she requested in advance (advance directives) but in other cases (e.g. a person in a vegetative state for years) requested by the family
4. *Palliative sedation* (formerly defined as *terminal sedation*): administration of medications with the intention to reduce refractory symptoms of suffering (e.g. opioids), even if the intervention may hasten the death of the patient (principle of double effect)

*For some authors, practices 1a and 2 can be subsumed under the rubric of Medical Assistance in Dying (MAiD) since in both physicians have a role in assisting the patient requesting to die

Gradually, starting from the Dutch experience dating almost 30 years back [1], these practices have been extended in some countries to people that are not terminally ill because of a somatic disease, but are affected by non-terminal conditions(MAiD-NT), including psychiatric disorders (e.g., depression, schizophrenia) or major neurocognitive disorders (e.g., dementia). The legislation in these jurisdictions is not identical, although some criteria are the same: the non-terminal condition should be refractory, resistant to treatment or untreatable, making the patients’ quality of life poor, and causing unbearable suffering (Table 2) [2•].

**Table 2:** Overview of legislations about MAiD-NT for people with psychiatric disorders in different countries

**Country**	**Law and year**	**Criteria**
**Euthanasia/physician-assisted suicide legal**		
Belgium	Euthanasia Law 2002	• Competent adults and emancipated minors (in 2014 euthanasia legalized for children) • The request is voluntary, well-considered and repeated, and is not the result of any external pressure • Medical condition of constant and unbearable physical or mental suffering that cannot be alleviated, resulting from a serious and incurable disorder caused by illness or accident • No specification on previous attempted treatments required to define the condition as “cannot be alleviated”
Netherlands	Termination of Life on Request and Assisted Suicide Act 2001	• Unbearable suffering without prospects of improvement (attempted treatments not specified, but every available and feasible treatment should be discussed thoroughly with the patient: whether an alternative treatment is available and feasible, there are prospects of improvement) • Voluntary request for E/PAS and persistent over time • Full awareness of his/her condition, prospects, and options • Independent physician should be consulted to grant for the request • Method used should be medically and technically appropriate • Age of at least 12 years old (parents’ consent required when aged between 12 and 16 years)
Luxembourg	Law on euthanasia and assisted suicide 2009	• Competent adults (16- to 18-year-olds need their parents or legal guardians consent) • Insufferable pain for a physic or psychic condition that can not be alleviated (attempted treatments not specified, but every available and feasible treatment should be discussed thoroughly with the patient, comprising palliative care) • Voluntary and repeated request • Written request
Canada	Medical Assistance in Dying Act 2016 *	• Canadian health insurance • Competent adults • A grievous and irremediable medical condition (“illness, disease, disability or state of decline that cannot be relieved under conditions that the patients consider acceptable”. No attempted treatment specified, but all available treatments, including palliative care, should be discussed) • Voluntary request for medical assistance in dying not resulting from outside pressure/influence
**Only PAS legal**		
Switzerland	1942	• Competent adults • The person has to perform him/herself the act • No selfish motive (if selfish motive for PAS, considered a crime)

*Revised as Bill C-7: as of March 2023, will include provision of MAiD for Mental Illness where the latter is sole source of suffering

The aims of the present critical review are (i) to examine and discuss the most significant issues related to this area, including the complexity of the definition of clear-cut and stringent criteria for and the moral issues involved in MAiD-NT when comparing patients with physical or mental disorders; (ii) to summarize the most relevant data concerning the problem of MAiD-NT among patients with psychiatric disorders, attitudes, and views of health care professionals, patients and caregivers, and epidemiological data.

## Methods

A search was made of the major databases over the last 10 years (Embase/Medline, PsycLIT, PsycINFO, the Cochrane Library) from January 2011 to June 2021, by including the most relevant studies in full journal articles investigating MAiD-NT in patients with psychiatric disorders. The literature search was performed with the search terms (euthanasia[title] OR “assisted suicide*”[title] OR “assisted dying”[title] OR “physician assistance in dying”[title]) AND (psych* OR mental) with appropriate filters (abstract, humans, English).

## Results

The analysis of the literature allowed us to extrapolate data on several topics, namely, the moral debate about MAiD-NT in psychiatry, the opinions, and attitudes about MAiD-NT in psychiatric settings, the epidemiological findings on MAiD-NT cases among patients with psychiatric disorders, and the impact of MAiD-NT on physicians.

### The Moral Debate About MAiD-NT in Psychiatry and Unsolved Problems

The moral debate in the case of MAiD-NT in psychiatry is motivated by the observation that many key aspects of these practices as applied in patients in a terminal phase of a somatic disorder are not pertinent in those with psychiatric disorders. For example, withdrawing/withholding treatment, even if not part of MAiD, does not apply to psychiatry where the intervention requires action and not omission. Action in fact relates to all the intentional activities (*doing*) that a physician may perform (such as in MAiD), while omission relates to the intentional decision to not act (*not doing or stop doing*). More specific moral issues in MAiD-NT are supporting death hastening practices for patients whose underlying condition invokes a desire to die when their desire to die can be reinforced by lack of support, opportunity, and access to resources that results from their marked marginalization.

When discussing opinions about MAiD-NT for people with mental illness, two opposite positions emerge as follows: those who are explicitly against those practices in patients with psychiatric disorders and those who support them as morally acceptable, with different arguments against and in favor of the practices (Table 3 and Supplement Table 1) [3••].

**Table 3:** Some arguments against and in favor of MAiD-NT in patients with psychiatric disorders

**Against**	**In favor**
• The duty of physicians is to preserve life, therefore to prevent suicide, not to cause it even if requested • Psychiatric disorders themselves can cause a desire to die • Depressive cognitive distortions can cause patients to see themselves, the world and the future as hopeless • Supporting a MAiD decision is a form of collusion with patients’ sense of helplessness • Severe psychiatric disorders (as well as dementia) may compromise the person's decision-making capacity, therefore the patient’s request is not valid • Pathophysiology of somatic disorders are clear, not the same for mental illness • Certainty that a person's psychiatric condition is untreatable is largely insufficient • Objectivity and quantification is not possible for psychiatric disorders vs somatic disorders • Uncontrollable pressures from family or society, including shortage of funds for health care, cause individual’s feelings of a duty to die, guilt about being alive, (that make institutionalisation of MAiD-NT for mental illnesses unacceptable) • MAiD-NT in psychiatric patients may lead to a slippery slope, extending to minors, old people with dementia; a way for vulnerable or marginalized persons to seek death as a relief from poverty, loneliness or other psychosocial stressors	• The duty of physicians is to reduce intolerable suffering as a form of care • Not all patients with severe mental illness are incompetent, their decision-capacity is not *always* compromised; therefore their possible request for MAiD-NT can be rational and valid • There is no difference in the suffering of patients with ‘typical’ terminal or futile medical disease and in those with severe psychiatric disorders • Prohibiting MAiD-NT infringes on patient’s rights, personal liberty; increases discrimination and stigma; and is an unacceptable attack on freedom and dignity • If the law is applied approprately, it should be able to distinguish between patients who are suicidal from those with a rational request to receive MAiD-NT. Therefore criteria of the law should be followed for each case and are a guarantee that MAiD-NT is not used as a form of legalized killing: • If under certain circumstances, death by omission (e.g. withholding/withdrawing treatment – which can be considered an actions since a physician acts when he/she interrupts tretaments) is moral, then death by action (e.g. MAiD-NT) is also moral • Leaving alone a patient when choosing suicide in a desperate way is immoral; also grief caused by a family member committing suicide is complicated and source of extreme suffering. Therefore it is necessary to help both patients and family for a dignified death by assisting him/her in suicide (if the criteria of law are met) and working in advance with the family

Regarding the first group who oppose the practices of MAiD-NT (and MAiD, in general), it is claimed that religious and secular traditions uphold the sanctity of human life and that physicians are called to act in accordance with the non-maleficence principle (*Primum non nocere*), avoiding by all means to damage their patients (Hippocratic oath). This group argues that medicine has to preserve human life and uphold suicide prevention. Since there is an important difference between passively “allowing to die” and actively “killing,” MAiD-NT is not morally justifiable. In this sense, medicine is defined by its devotion to a clearly stated purpose: healing and the conservation of health and life. Therefore, in the doctor-patient relationship stands the moral duty to preserve “the most intimate, most personal, and most humane uses of technology—the helping, caring, and curing of vulnerable, anxious, dependent, and trusting members of the human community” [4] (page 24).

As a further argument, its is said that all people have obligations (e.g., to their family and society in general), until their death. These obligations limit their rights. While patients may refuse treatment, they do not have the right to be killed, since this, in the reciprocity of the doctor-patient relationship, would mean that physicians have a duty to kill [5].

Another issue is determining an objective prognosis in mental illnesses versus physical illnesses. For example, whether a disorder is irremediable in a somatic disease (e.g., advanced stage of cancer) is quite clear, it is not so clear in mental disorders, since irremediability is difficult to be predicted in MAiD-NT [6••]. With respect to this, Gaind [7•]  poses a series of questions  “[..] would society consider a (any number)% chance of irremediability from advancing metastatic cancer to be the same as a (same number)% chance of irremediability of suffering from a clinical depression associated with significant loneliness and poverty?”; or also “Given the absence of any guidance regarding predicting irremediability, does this mean that it would be discriminatory to not provide MAiD to patients for mental illness, or that it would be discriminatory to provide such patients MAiD by exposing them to death based on unscientific determinations of unpredictable irremediability for which there are no standards?” (pp. 604–606). In this sense with regard to the issue of irremediablity, MAiD-NT practices lack evidence-based data and without those data, as in all the branches of medicine, it is a risk to apply the practices whatever their aims are [8].

Also, risks have been raised regarding the “slippery slope” phenomenon, meaning that the extension of MAiD from terminal illness to other conditions, may facilitate losing control of the practice. For example, economic pressures are a reality in most health systems, with the risk that providing MAiD is far more cost-effective than medical care to chronically ill patients [9] Furthermore, some of the people requesting MAiD-NT are likely to be a source of usable organs for transplantation. Strengthening the link between MAiD and organ donation could damage the trust in medical, professional and public health authorities [10]. Therefore, MAiD-NT is extremely dangerous, especially when extended to minors and people with psychiatric disorders, as well as in those whose cognitive impairments (e.g. dementia) make it difficult to assess decisional capacity [11, 12]. The risk is that society could have the right to get rid of people with problems that require considerable resources, hypocritically masking it as a humanitarian act to be performed for the patient’s own good [13–16].

In an opposite position are those who consider MAiD-NT morally permissible, thus endorsing the extension of those practices to patients with psychiatric disorders. Proponents of this view argue that the single individual (neither the society nor the family) is the owner of his/her own life (including death, as part of life process). Thus, as an autonomous, rational, and self-aware individual, his/her decisions, including those about time and circumstances of death, should be respected (e.g., [17, 18]). On this basis, giving everybody the right to have a good death through MAiD-NT should be acceptable as a universal principle, since prolonging an unacceptable or unbearable life, marked by agony and loss of dignity, is immoral. On these premises, prohibiting MAiD-NT limits the rights of personal liberty and prevents intervention aimed at solving loss of independence, sense of purpose and meaning, and functional capacities. These argumentations suggest that we need to understand that life is not always the best outcome, and death is not always the worst, as death can be a better alternative than living in the presence of suffering (utilitarian perspective). Also, if an action, such as MAiD-NT, promotes the best interest of everyone concerned and violates no one’s rights, then that action is morally acceptable (libertarian perspective). In this way, the Hippocratic Oath’s directive that “I will take care that they (the sick) suffer no hurt or damage” should be interpreted as the duty for physicians to help a person to avoid prolonged suffering, including ending the person’s life, as a form of care.

A further argument supporting MAiD-NT is to dissolve the distinction between deaths caused by actions and death caused by omissions or inaction. If no morally significant difference can be found between deaths caused by omissions or by actions, then, by extension, there are grounds for allowing for death caused by actions. In this sense, Rachels [19] argues that letting a person die is a type of action, demonstrated by the fact that we would consider a doctor blameworthy if he needlessly let a person die.

It is however true that some who consider MAiD acceptable for people in a terminal somatic condition face a series of challenges in people with mental illness as follows: the ethical distinction between helping a dying person die peacefully versus providing death to a non-dying person; the unpredictability of prognosis of non-terminal conditions such as most mental illnesses; the difficulty that MAiD-NT may put vulnerable or marginalized people at risk of seeking death as a relief from poverty, loneliness, or other psychosocial stressors [10, 20–24]. For example, Trachsel and Jox [25••] examined the criteria for MAiD-NT in Switzerland and considered the criteria put forward by the Swiss Academy of Medical Sciences, namely, intolerable suffering due to severe illness or functional limitations (and acknowledged as such by a physician), are not sufficient. The authors, instead, underline that suffering is a necessary but not sufficient condition and that decision-making capacity and refractoriness of suffering should also be included as key criteria.  Moreover, they contend that suffering, as a subjective experience, can only be quantified by the patient and not be objectively compared across individuals. This means that “intolerable suffering” is different across individuals and that it should encompass perceived burdensomeness i.e. the perception that one is a burden or drain on significant others or a burden on society.

On the other side, although it is clear that MAiD-NT requires careful safeguards to be permitted in people with refractory mental illness, to exclude all individuals requesting MAiD-NT falsely implies that everyone in that category lacks capacity. In fact, apart from those who are legally declared incompetent, few patients with psychiatric disorders lack capacity (e.g., 4% of patients with a personality disorder, 80% of patients with acute psychosis due to schizophrenia, 30% of patients with major depression) [26••].

### Opinions and Attitudes About MAiD-NT for People with Psychiatric Disorders

Linked with the moral debate are the opinions that physicians (or health care professionals) and patients and their caregivers have about MAiD-NT (Supplement Table 2 for details).

### Opinions and Attitudes of the General Population

Data are available from studies examining general population opinions about MAiD-NT, with significant differences between countries where the practices are legalized and those where the practices are prohibited.

For example, in the Netherlands, half of the respondents (53%) of the general population supported the eligibility of psychiatric patients for MAiD-NT, with only 15% stating their opposition to this practice [27]. In contrast, among the US general population, one-third supported legalizing MAiD-NT for people with physical disability (e.g., cerebral palsy) and dementia or mental illness (e.g., depression) [28]. This percentage significantly decreased, however, when a series of scenarios were presented showing the possible request by patients in non-terminal state because of loss of support from health care system or poverty [29].

### Opinions and Attitudes of People with Psychiatric Disorders

Studies specifically examining psychiatric patients’ opinions on MAiD-NT are lacking. Having resistant or refractory psychiatric disorders can cause unbearable suffering—which has been defined as “a profoundly personal experience of an actual or perceived impending threat to the integrity of life of the person, which has a significant duration and a central place in the person’s mind” [30]. However, to fully understand what unbearable mental suffering is for patients with psychiatric disorders, directly questioning those who perceived it is mandatory. Analysis of requests for MAiD-NT by 26 Belgian psychiatric patients indicated five main categories of suffering: medically related, intrapersonal, interpersonal, societal-related, and existential suffering [31]. A Dutch study showed that the most frequent reasons for MAiD-NT were irreversibility, loss of control, emptiness and emotional flooding, freezing, social distancing, narcissistic wounds, confusion, and self-estrangement [32]. A further study showed that patients with psychiatric disorders reported autonomy and self-determination, ending the suffering, recognition as being persons, and dignified end-of-life as the main reasons to request MAiD-NT [33].

### Opinions and Attitudes of Relatives of Patients with Psychiatric Disorders

While there is literature about MAiD examining caregivers and family relatives of patients with terminal somatic illness [34], there are only very few studies on families of patients with psychiatric disorders. A qualitative Dutch study showed that although the relatives of psychiatric patients were understanding of their wish to die (e.g., compassion, respect for autonomy, fear that the patient will commit a gruesome suicide), they also hoped that they would make another choice [35].

Regarding the outcome, people bereaved by MAiD (but not MAiD-NT) generally had similar or lower scores on measures of disordered grief, mental health, and posttraumatic stress compared with those who died naturally [36]. However, a Swiss study [37] showed that among members or close friends who were present at an assisted suicide, 13% met the criteria for full Post Traumatic Stress Disorder (PTSD), 6.5% for subthreshold PTSD, and 4.9% for complicated grief, indicating a higher prevalence of psychiatric disorders than the general population. However, specific data on family reactions after MAiD-NT for a patient with psychiatric conditions are not available.

### Opinions and Attitudes of Physicians

Regarding the opinions of physicians on MAiD-NT, there are also great variations. In countries where the practice is legalized, physicians of different specialties generally endorse MAiD-NT, with differences according to the medical specialty (Supplement Table 2 for details). In the Netherlands, 20% of medical specialists endorsed the practice, about half among general practitioners (main reasons: responsibility, self-determination, compassion, fairness, and preventing suicide) [27, 38]. Early and advanced stages of dementia are considered less conceivable conditions than patients with terminal somatic diseases [39]. However, a large survey of more than 2,000 physicians showed that while the majority would grant a request for MAiD in somatically ill patients, only about one-third found MAiD-NT conceivable, and one-quarter endorsed it for people just tired of living [40].

Regarding mental health care professionals, including psychiatrists and psychiatric nurses, a specific point is that these individuals are usually trained to address suicidal ideation and behavior, by emphasizing hopes and purpose for living and by treating as much as possible the underlying disorders. Therefore, they are particularly aware of the dilemma caused by MAiD-NT. However, in the cited Dutch study [41, 42], about 2/3 of the psychiatrists believed it is possible to establish whether a psychiatric patient’s suffering is unbearable. The majority (74.5%) felt MAiD-NT should remain legal for adult psychiatric patients, who are experiencing unbearable mental suffering, who are able to make a well-considered request, and whose situation is hopeless. Half of them agree that MAiD-NT is compatible with a psychiatric care relationship [43] in which it is possible to establish whether the desire to die is entangled with a psychiatric disorder [41]. Dutch psychiatric nurses are also supportive of patients with different mental disorders to make an informed decision about MAiD-NT [44] and the majority feel themselves able to address and discuss the issue with their patients [45]. Similar data were reported in Switzerland [46].

In Canada, only one-third support MAiD-NT for mental illnesses [47, 48], with low endorsement related to the risk of changing the psychiatrists’ commitment to their patients; having a personal faith; and having had patients who would have received MAiD-NT for mental illness but who recovered. Cultural factors may also play a role, as shown in Israel, where psychiatrists displayed more conservative views on MAiD-NT for mental disorders than physicians from other medical specialties [49].

### Attitudes Versus Active Role

Opinions about the practices of MAiD-NT do not necessarily correspond to playing a direct role. In fact, despite the support of Belgian psychiatrists for MAiD-NT, only a minority (8.4%) would actually engage in performing it with their own patients, and even less would perform MAiD-NT for a colleague’s patient (4.5%) [43]. Furthermore, although about 80% psychiatrists in Belgium had to deal with at least one MAiD-NT request, only 5% were actually involved in the administration of lethal drugs or attended when another physician was performing the act for their own patient [50].

The first point here is to determine if the criteria for MAiD-NT for patients with psychiatric disorders are equivalent to patients with somatic terminal conditions, given that suicide ideation can be a symptom of mental disorders. This point speaks to the 2-track approach of the Dutch law, where mental health care professionals have to evaluate the possible MAiD request from their patients to grant the request itself, while at the same time, maintaining a recovery-oriented care and being committed to suicide prevention [51, 52].

A second point is the difference between irrational and rational suicidality, with one position suggesting that suicide is always an irrational act and the other supporting the view that patients who suffer from a severe mental disorder but maintain competence, such as severe depressive disorders or schizophrenia, can indeed have a rational wish to die [53–55, 56••, 57].

There are other motives regarding healthcare professional involvement or non-involvement in MAiD-NT, such as lack of criteria or difficulties in applying these criteria [50, 58] or the problem of suicide in those who had their request refused [59].

### Studies on MAiD-NT Cases Among People with Psychiatric Disorders

Analysis of the literature of the last years shows a constant increase of MAiD-NT cases and requests in the context of patients with psychiatric disorders (Supplement Table 3).

In Belgium, MAiD-NT due to psychiatric disorder have increased from 0.5% of all cases reported in the period 2002–2007 to 3.0% of all cases reported in 2011, [60] with a total number of psychiatric patients explicitly requesting it between 1,100 and 1,150 (2015–2016) and 60–70 patients effectively receiving it. The requests due to psychiatric disorders, mainly depression and personality disorders, in young patients, became the sixth most common indication. In most cases, patients were less than 70 years old (mean age 47 years) [61] and about half of patients had made suicide attempts before [62, 63], including  while waiting for approval [61].

In the Netherlands, the number of MAiD-NT cases increased from 2011 (*n* = 2) to 2017 (*n* = 83) [64], for patients with mood or personality disorders, mainly women and aged between 50 and 70 years [62, 65].

In Canada, where the Supreme Court extended MAiD to other conditions beyond terminal illnesses (to “a person whose natural death is not reasonably foreseeable”) [66], mental illnesses are temporarily excluded from eligibility up until March 2023. In the meantime, the Minister of Justice and Health will provide guidelines for MAiD-NT. A series of cases of patients with psychiatric disorders who applied for MAiD-NT were denied it, although, based on their characteristics, would have been eligible for MAiD-NT in other countries (e.g. Belgium, Netherlands) [67, 68]. The number of patients with borderline personality disorders requesting MAiD-NT is also markedly increasing [69••].

### The Impact of MAiD-NT in Mental Health Professionals

There is a dearth of research on how MAiD in general may impact physician health and well-being, but even less is known about MAiD-NT. The uncertainty of predicting irremediability, possible conflation of psychiatric suffering with illness suffering, and the challenges of differentiating patients seeking MAiD-NT from the suicidal patients will all likely impact health care professionals [70]. For example, there are data showing that advance request for MAiD-NT in cases of patients with dementia was complicated by ambiguous directives, patients being unaware of the procedure, and physicians’ difficulty in assessing “unbearable suffering.” [71]

For some disorders, such as borderline personality disorders, a physician intervening by MAiD-NT could encounter moral distress, since the criteria for the procedure might not take into consideration the common fluctuating suicidal ideation and behavior in these disorders, the contemporary forms of psychosocial rehabilitation treatment, and the individual’s potential for having a life worth living [68, 72].

## Conclusion

The increasing number of countries endorsing and legalizing MAiD-NT for people with psychiatric disorders underscores the need to examine this phenomenon in more detail and from many different perspectives.

The first moral issue we have summarized indicates that criteria for MAiD-NT in people with intolerable suffering caused by non-terminal disease, such patients with psychiatric disorders (but also with neurological disorders refractory to any treatment, dementia, or even minors or people with existential suffering), are more problematic than the criteria for patients with somatic incurable diseases in an advanced or terminal phase. This is a moral dilemma that can cause significant conflict: on the one hand, it can be argued that professional roles, responsibilities, and personal expectations include the duty to preserve life, and that failure to do so by way of not having foreseen and prevented suicide can result in moral and legal culpability. On the other hand, there is the professional obligation to help and not abandon patients and the duty to relieve suffering, by helping them to die. There also needs to be a better definition and ways to assess unbearable suffering that patients’ experiencing psychiatric disorders, and further exploration of how this can be addressed in the therapeutic setting [73].

A second moral issue regards the “rationality” of a request to be helped in dying. Although physicians differentiate three types of death wishes among patients with psychiatric disorders (death wish related to psychopathology or *impulsive suicidality*; death wish consistent over time, or *chronic suicidality*, and *rational death wishes*) [74], more research should be carried out in this area.

A third issue we have addressed is the impact of MAiD-NT in physicians. In spite of the importance of this topic, it has been largely neglected with only a few studies examining the impact of MAiD on healthcare professionals in palliative care setting. Thirty to fifty percent of physicians expressed emotional burden or discomfort regarding participation, and adverse personal impact in 15–20% [75•]. The risk for health care professional to suffer and become psychologically vulnerable because of emotional exhaustion, detachment toward patients, poor professional accomplishment, moral distress, or even depression and risk of suicide should be considered when examining the implications of MAiD-NT [76].

In summary, although the literature has shown that a small percentage of people with psychiatric disorders died by MAiD-NT in comparison with patients with somatic terminal diseases, the problems it elicits are not solved yet. How to make objective criterion of irremediability of a mental disorder; how to balance suicide prevention with assisted suicide; how to avoid the risk of progressively including in MAiD-NT requests by vulnerable segments of the population, such as minors and elderly or people with dementia, in a productive-oriented society, are some of the critical points to be considered. As indicated from the beginning in the Dutch experience [77, 78], the application of MAiD-NT in people with mental disorders should be further explored, to prevent end-of-life rights from contradicting the principles of recovery-oriented care in psychiatry.

## Supplementary Information

Below is the link to the electronic supplementary material.

Supplementary file1 (DOCX 38 KB)

Supplementary file2 (DOCX 46 KB)

Supplementary file3 (DOCX 32 KB)
