# Supplementary material for: Debating Euthanasia and Physician-Assisted Death in People with Psychiatric Disorders
Source: Curr Psychiatry Rep. 2022 Jun 9;24(6):325–35. doi: 10.1007/s11920-022-01339-y (PMC9203391; doi:10.1007/s11920-022-01339-y)
Supplement: Supplementary file 1 — Supplementary file1 (DOCX 38 KB) [file 11920_2022_1339_MOESM1_ESM.docx]

**Supplement Table 1. Studies about ethical issues in MAiD-NT**

| **Ethical issues** | | | |
| --- | --- | --- | --- |
| Berghmans et al., 2013 | Netherlands | Case report | Objection against offering E/PAS to patients suffering from mental illness: offering E/PAS reinforces loss of hope and demoralization. |
| Cholbi, 2013 | U.S.A. | Review on the notions of unbearable suffering, futility, and terminality | A psychiatric disorder is not per se a disqualifier: seeking assisted suicide due to psychiatric disorders should face no barriers greater than those with physical Illness |
| Varelius, 2016 | Finland | Opinion article on limiting E/PAS to autonomous  or rational psychiatric patients | The author distinguishes between   - Involuntary euthanasia: patient put to death against her will - Non-voluntary euthanasia: patient incapable of having desires about dying. - Voluntary euthanasia: autonomous desider to die.   The author argues that only autonomous requests of E/PAS could be morally acceptable, that is when it is voluntary, intentional, and based on sufficient understanding about its nature and consequences. Despite being relevant for E/PAS, psychiatric suffering is not necessary for the moral acceptability of E/PAS. |
| Doernberg et al., 2016 | Netherlands | Review of psychiatric EAS case summaries (66 cases 2011-2014).  Content analysis on texts discussing capacity. | Capacity distincted in two domains:   - Capacity-specific abilities - Clinical features related to capacity   Usually physicians rely on the absence/presence of clinical symptoms to assess capacity.  55% only global judgments of patients’ capacity, no reference to specific capacity criteria.  32% assessment of capacity-specific abilities.  12% disagreement between physicians on patient capacity |
| Blikshavn et al., 2017 | Norway | Arguments against E/PAS in TRD | Why E/PAS should not be offered for TRD : TRD as a psychopharmacological concept ; individual prognosis not equal to group prognosis ; giving up of hope ; normalization of E/PAS as a risk for mental health practice. |
| Mendz and Kissane, 2020 | Australia | Review on the concepts of agency and decisional capacity | There’s a need to assess the agency of individuals, particularly in life or death circumstances, in order to respect properly their personal autonomy. |
| van Veen et al., 2020 | Netherlands | Case report | Case of a patient with schizophrenia and refractory musical hallucinations who requested E/PAS. A second opinion recognized his symptoms as intrusive thoughts and the patient recovered through an appropriate treatment.  This case arises reflections on uncertainty in psychiatric illness and need for adequate procedures. |
| van Veen et al., 2020 ** | Netherlands | Review on 50 articles on irremediability and E/PAS for psychiatric patients | Three arguments concerning irremediability   1. *Uncertainty* about irremediability is inevitable : what level of certainty would be needed for E/PAS in psychiatry to be acceptable ? 2. *Hope*. More research needed on relationship between hope and E/PAS requests in psychiatric patients. 3. *Treatment* *refusal* in search of E/PAS : more research needed on kind of treatments and motive for refusal |
| Nicolini et al., 2020 ** | Belgium | Systematic review of 42 papers on reasons for and against E/PAS | 8 domains :  1) mental and physical illness and suffering  2) decisional capacity  3) irremediability  4) goals of medicine and psychiatry  5) consequences for mental health care  6) psychiatric E/PAS and suicide  7) self-determination and authenticity  8) psychiatric E/PAS and refusal of life-sustaining treatment  Mostly non-clinicians argued for parity between mental and physical illness and that E/PAS laws on physical illness should be extended to include mental illness.  Disagreements were based on non parity between physical and psychiatric illnesses and questioning the prognosis prediction in psychiatry. |
| Clarke et al., 2021 | Ireland | Considerations on E/PAS for psychiatric patients | The authors examine issues on E/PAS in psychiatric patients after the proposal of the Dying with Dignity Bill, 2020   1. *Concept of terminal illness*. Despite definite criteria may be set, this line could be revisited over time. Moreover, discrimination could arise when allowing E/PAS in some settings and not others. 2. *Capacity and voluntariness.* Assessment of capacity not clearly specified. 3. *Clear and settled intention.* No clear definition as it could be a symptom of the psychiatric illness and may change over time. 4. *Euthanasia and suicide.* Raises the question : who gets suicide prevention and who gets suicide assistance ? 5. *Psychological suffering and palliative care.* Issues on personal experience of suffering and inadequate palliative care systems. 6. *Effects on the therapeutic relationship.* 7. *Autonomy and conscientious objection.* The referring of patients to other physician for E/PAS could be viewed as collusion. |
| Nicolini et al., 2022 | Netherlands, Belgium | Debate on E/PAS and suicide prevention considering gender gap | Gender paradox in suicide (men prevalent) and E/PAS (women prevalent) related to capability, arising considerations on possibility of completing suicide through E/PAS and risk for avoidable deaths. Clearer clinical guidances are needed, with a particular attention towards women. |
| Brodeur et al., 2022 |  |  | Criteria for granting MAiD-NT request in patients with borderline personality disorders are complex. Is BPD a cause of enduring and intolerable suffering which is irremediable and cannot improve over time, when clinical experience indicate that almost the totality of patients improve their functions and their symptoms? Are patients with BPD not impaired in providing informed consent or in making rational decisions, considering that impulsivity, attentional biases toward negative stimuli, and thought distortions regarding the nature of the world, are typical of BPD? |

E/PAS : Euthanasia/Physician Assisted Suicide ; UMS : Unbearable Mental Suffering ; TRD : Treatment Resistant Depression

**References**

Berghmans, R., Widdershoven, G., Widdershoven-Heerding, I., 2013. Physician-assisted suicide in psychiatry and loss of hope. Int. J. Law Psychiatry 36, 436–443. https://doi.org/10.1016/j.ijlp.2013.06.020

Blikshavn, T., Husum, T.L., Magelssen, M., 2017. Four Reasons Why Assisted Dying Should Not Be Offered for Depression. J. Bioeth. Inq. 14, 151–157. https://doi.org/10.1007/s11673-016-9759-4

Brodeur, J., Links, P.S., Boursiquot, P.-E., Snelgrove, N., 2022. Medical Assistance in Dying for Patients with Borderline Personality Disorder: Considerations and Concerns. Can. J. Psychiatry 67, 16–20. https://doi.org/10.1177/0706743721993645

Cholbi, M.J., 2013. The terminal, the futile, and the psychiatrically disordered. Int. J. Law Psychiatry 36, 498–505. https://doi.org/10.1016/j.ijlp.2013.06.011

Clarke, C., Cannon, M., Skokauskas, N., Twomey, P., 2021. The debate about physician assisted suicide and euthanasia in Ireland – Implications for psychiatry. Int. J. Law Psychiatry 79, 101747. https://doi.org/10.1016/j.ijlp.2021.101747

Doernberg, S.N., Peteet, J.R., Kim, S.Y.H., 2016. Capacity Evaluations of Psychiatric Patients Requesting Assisted Death in the Netherlands. Psychosomatics 57, 556–565. https://doi.org/10.1016/j.psym.2016.06.005

Mendz, G.L., Kissane, D.W., 2020. Agency, Autonomy and Euthanasia. J. Law, Med. Ethics 48, 555–564. https://doi.org/10.1177/1073110520958881

Nicolini, M.E., Gastmans, C., Kim, S.Y.H., 2022. Psychiatric euthanasia, suicide and the role of gender. Br. J. Psychiatry. https://doi.org/10.1192/bjp.2021.95

Nicolini, M.E., Nicolini, M.E., Kim, S.Y.H., Churchill, M.E., Gastmans, C., 2020. Should euthanasia and assisted suicide for psychiatric disorders be permitted? A systematic review of reasons. Psychol. Med. 50, 1241–1256. https://doi.org/10.1017/S0033291720001543

van Veen, Sisco M.P., Ruissen, A.M., Widdershoven, G.A.M., 2020. Irremediable Psychiatric Suffering in the Context of Physician-assisted Death: A Scoping Review of Arguments: La souffrance psychiatrique irrémédiable dans le contexte du suicide assisté : Une revue étendue des arguments. Can. J. Psychiatry 65, 593–603. https://doi.org/10.1177/0706743720923072

van Veen, S. M.P., Scheurleer, W.F.J., Ruijsch, M.L., Röder, C.H., Widdershoven, G.A.M., Batalla, A., 2020. Last-minute recovery of a psychiatric patient requesting physician-assisted death. Psychiatr. Serv. 71, 621–623. https://doi.org/10.1176/appi.ps.201900489

Varelius, J., 2016. On the Moral Acceptability of Physician-Assisted Dying for Non-Autonomous Psychiatric Patients. Bioethics 30, 227–233. https://doi.org/10.1111/bioe.12182
