# Supplementary material for: Debating Euthanasia and Physician-Assisted Death in People with Psychiatric Disorders
Source: Curr Psychiatry Rep. 2022 Jun 9;24(6):325–35. doi: 10.1007/s11920-022-01339-y (PMC9203391; doi:10.1007/s11920-022-01339-y)
Supplement: Supplementary file 2 — Supplementary file2 (DOCX 46 KB) [file 11920_2022_1339_MOESM2_ESM.docx]

**Supplement Table 2. Data and opinions about MAiD-NT among physicians, general population and patients with psychiatric disorders**

| **Reference** | **Country** | **Type of study** | **Results** |
| --- | --- | --- | --- |
| Levy et al., 2013 | Israel | Cross sectional questionnaires to 49 psychiatrists and 54 physicians | More conservative views in religious psychiatrists than other physicians, in female physicians and orthodox physicians. |
| De Hert et al., 2015 | Belgium | Multicenter survey in psychiatric nurses | Need for ethically sound and comprehensive provision of care and underline an important role of psychiatric nurses in dealing with the issue of request for euthanasia.  The majority of participants did not object euthanasia based on unbearable mental suffering. A minority stated that euthanasia should be restricted only to physical suffering.  19 participants found euthanasia ethically unacceptable. |
| Bolt et al., 2015 | Netherlands | Cross-sectional survey among 1456 physicians providing care for patients (no psychiatrist) | Physicians who would grant a request for patients with:   - Cancer or physical disease 85% and 82% - Psychiatric disease 34% - Early-stage dementia 40% - Advanced dementia 29-33% - Tired of living 27% |
| Rousseau et al, 2017 | Canada | Cross-sectional survey among 528 Canadian psychiatrists | Psychiatrists who supported MAiD-NT on the basis of mental illness in some circumstances = 29.4%  Factors correlating with decreased support for MAiD-NT for mental illness: belief that MAiD-NT would change the psychiatrists' commitment to their patients through enduring suffering; having a personal faith; having had past patients who would have received MAiD-NT for mental illness were it legal but instead went on to recover. |
| Verhofstadt et al., 2017 | Belgium | Testimonials (self-recorded or self-written) from 26 psychiatric patients who requested euthanasia | 5 domains of suffering identified :   1. *Medical/somatic* : somatic symptoms, cognitive impaiment/complaints. However, psychic symptoms perceived as worse than medical ones. 2. *Intrapersonal* : traumas, self-destructive thoughts. 3. *Interpersonal* : conflicts or disruptions with important others, irreparable losses, lack or loss of social support or understanding from important others, witholding information from important others, less social contacts. 4. *Societal* : pressure to make a living, financial problems and health insurance problems, low income, work-related issues, disability, behavioural adjustment problems to society, feeling a burden to society, but also blaming society, social isolation. 5. *Existential* : feelings of being overwhelmed by an existential fear of life without finding any quality of life, lack or loss of control over the disorder, symbolic death of patient’s self or self-representation.   Hopelessness was confirmed to be an important contributor. |
| Demedts et al., 2018 | Belgium | Cross sectional survey March-April 2014  N=133 mental health nurses | 52% nurses had been involved, at least once, in the process of UMS euthanasia  3/4 endorsed that psychiatric patients can make well-considered requests regarding UMS euthanasia and that this is not part of their disease  A large proportion of nurses supported that a patient with a psychotic disorder (83%), a resistant depression (71%), or a bipolar disorder (70%) is able to make an informed decision about UMS euthanasia.  Majority of nurses perceived lack of information and guidelines regarding UMS euthanasia |
| Pronk et al., 2019 | Netherlands | 17 interviews with psychiatrists  January-June 2016 | Reasons considered for and against E/PAS : moral, epistemological, practical and contextual.  Changes in views on E/PAS in psychiatry related to changes in the view on the nature of psychiatric disease : some psychiatrists recognize parity between physical and mental illness, others stress their differences. |
| Hetzler et al., 2019 | U.S.A. | Cross sectional survey to 1.000 US physicians | 60% of physicians thought PAS should be legal, 38% illegal and 69% thought it should be decriminalized, while 30% thought it should not decriminalized in their respective states |
| Evenblij et al., 2019a | Netherlands | Cross sectional survey May-September 2016  500 psychiatrists (response rate: 49%) | 1100-1150 estimated patients requested E/PAS from 2015 to 2016  Approximately 6% patients received E/PAS in this period.  Most common reasons for accepting E/PAS: “suffering without prospect of improvement” (N=5), “feelings of depression”(N=5), “desperate situations in several areas of life” (N=3) “No longer being able to live independently” (N=3) |
| Evenblij et al., 2019b | Netherlands | Online questionnaire for general public (N=1965)  Written questionnaire for physicians (N=1374)  May-September 2016 | General public: 53% in favour for E/PAS in psychiatric patients.  Physicians : 20% medical specialists and 47% general practitioners in favour for E/PAS for psychiatric patients. Less agreement among women, religious individuals, medical specialists and psychiatrists. |
| Lengvenyte et al., 2020 | Netherlands | Exploratory retrospective content analysis of 66 digital cases of individuals who died by psychiatric EAS in the Netherlands between 2011 and 2014 | Psychological pain dimension:  Irreversibility: 100%  Loss of control: 83.3%  Emptiness: 63.6%  Emotional flooding: 59.1%  Freezing: 47%  Social distancing: 42.4%  Narcissistic wounds: 31.8%  Confusion: 24.2%  Self-estrangement: 18.2%  Multidimensionality of psychological pain in individuals who were granted EAS |
| Verhofstadt et al., 2020a | Belgium | Cross sectional survey  499 eligible psychiatrists (response rate: 40.2%) | 74.5% agree that euthanasia should remain possible for psychiatric patients  No significant associations between psychiatrist’s attitude to E/PAS remaining legal in psychiatry and their characteristics in terms of sex, perceived competence, work setting and work experience  Years of work experience and older age were positively associated with not considering an active role  Fewer years of work experience and a younger age were positively associated with referring the patient to a colleague for the clarification of the E/PAS request  Different ranges in years of work experiences were also statistically significant in considering an active role as preliminary advising physician |
| Verhofstadt et al., 2021a | Belgium | Cross sectional survey to 753 psychiatrists  November 2018-April 2019 | E/PAS assessment procedures may require months/years  Psychiatrists perceive challenges in assessment, especially in evaluating and fulfilling legal criteria  Main reason for request was existential suffering |
| Verhofstadt et al., 2021b | Belgium | Interviews with 16 psychiatric patients requesting E/PAS August 2019-July 2020 | Thematic coding of the interviews displayed the following main themes :   - Burden of the medical condition and long history of the disease - Adverse life events - Negative self-perception - Problems in interpersonal relationships - Societal challenges   Among the motives for E/PAS request, the perception of E/PAS as a therapeutic tool to restore hope and meaning to life.  Motives for E/PAS preference than self-suicide : rational evaluation, avoids trauma for family/caregivers, support from loved ones, avoids legal implications, but self-suicide as last resort if E/PAS denied |

**References**

Bolt, E.E., Snijdewind, M.C., Willems, D.L., van der Heide, A., Onwuteaka-Philipsen, B.D., 2015. Can physicians conceive of performing euthanasia in case of psychiatric disease, dementia or being tired of living? J. Med. Ethics 41, 592–598. https://doi.org/10.1136/medethics-2014-102150

De Hert, M., Van Bos, L., Sweers, K., Wampers, M., De Lepeleire, J., Correll, C.U., 2015. Attitudes of psychiatric nurses about the request for euthanasia on the basis of unbearable mental suffering (UMS). PLoS One 10, 1–13. https://doi.org/10.1371/journal.pone.0144749

Demedts, D., Roelands, M., Libbrecht, J., Bilsen, J., 2018. The attitudes, role &amp; knowledge of mental health nurses towards euthanasia because of unbearable mental suffering in Belgium: A pilot study. J. Psychiatr. Ment. Health Nurs. 25. https://doi.org/10.1111/jpm.12475

Evenblij, K., Pasman, H.R.W., Pronk, R., Onwuteaka-Philipsen, B.D., 2019a. Euthanasia and physician-assisted suicide in patients suffering from psychiatric disorders: A cross-sectional study exploring the experiences of Dutch psychiatrists. BMC Psychiatry 19, 1–10. https://doi.org/10.1186/s12888-019-2053-3

Evenblij, K., Pasman, H.R.W., Van Der Heide, A., Van Delden, J.J.M., Onwuteaka-Philipsen, B.D., 2019b. Public and physicians’ support for euthanasia in people suffering from psychiatric disorders: A cross-sectional survey study. BMC Med. Ethics 20, 1–10. https://doi.org/10.1186/s12910-019-0404-8

Hetzler, P.T., Nie, J., Zhou, A., Dugdale, L.S., 2019. A report of physicians’ beliefs about physician-assisted suicide: A national study. Yale J. Biol. Med. 92, 575–585.

Lengvenyte, A., Strumila, R., Courtet, P., Kim, S.Y.H., Olié, E., 2020. “Nothing Hurts Less Than Being Dead”: Psychological Pain in Case Descriptions of Psychiatric Euthanasia and Assisted Suicide from the Netherlands. Can. J. Psychiatry 65, 612–620. https://doi.org/10.1177/0706743720931237

Levy, T.B., Azar, S., Huberfeld, R., Siegel, A.M., Strous, R.D., 2013. Attitudes towards euthanasia and assisted suicide: A comparison between psychiatrists and other physicians. Bioethics 27, 402–408. https://doi.org/10.1111/j.1467-8519.2012.01968.x

Pronk, R., Evenblij, K., Willems, D.L., van de Vathorst, S., 2019. Considerations by Dutch psychiatrists regarding euthanasia and physician-assisted suicide in psychiatry: A qualitative study. J. Clin. Psychiatry 80. https://doi.org/10.4088/JCP.19m12736

Rousseau S, Turner S, Chochinov HM, Enns MW, Sareen J. A National Survey of Canadian Psychiatrists' Attitudes toward Medical Assistance in Death. Can J Psychiatry. 2017;62(11):787-794.

Verhofstadt, M., Audenaert, K., Van den Broeck, K., Deliens, L., Mortier, F., Titeca, K., De Bacquer, D., Chambaere, K., 2021a. Euthanasia in adults with psychiatric conditions: A descriptive study of the experiences of Belgian psychiatrists. Sci. Prog. 104. https://doi.org/10.1177/00368504211029775

Verhofstadt, M., Audenaert, K., Van Den Broeck, K., Deliens, L., Mortier, F., Titeca, K., Pardon, K., Chambaere, K., 2020. Belgian psychiatrists’ attitudes towards, and readiness to engage in, euthanasia assessment procedures with adults with psychiatric conditions: A survey. BMC Psychiatry 20, 1–10. https://doi.org/10.1186/s12888-020-02775-x

Verhofstadt, M., Pardon, K., Audenaert, K., Deliens, L., Mortier, F., Liégeois, A., Chambaere, K., 2021b. Why adults with psychiatric conditions request euthanasia: A qualitative interview study of life experiences, motives and preventive factors. J. Psychiatr. Res. 144, 158–167. https://doi.org/10.1016/j.jpsychires.2021.09.032

Verhofstadt, M., Thienpont, L., Peters, G.-J.Y., 2017. When unbearable suffering incites psychiatric patients to request euthanasia: qualitative study. Br. J. Psychiatry 211. https://doi.org/10.1192/bjp.bp.117.199331
