# Supplementary material for: Debating Euthanasia and Physician-Assisted Death in People with Psychiatric Disorders
Source: Curr Psychiatry Rep. 2022 Jun 9;24(6):325–35. doi: 10.1007/s11920-022-01339-y (PMC9203391; doi:10.1007/s11920-022-01339-y)
Supplement: Supplementary file 3 — Supplementary file3 (DOCX 32 KB) [file 11920_2022_1339_MOESM3_ESM.docx]

**Supplement Table 3. Reviews of studies on MAiD-NT in psychiatry**

| Thienpont et al., 2015 | Belgium | Retrospective analysis of outpatient psychiatric clinical record 2007-2011  Follow up in 2012  N=100 psychiatric patients requesting E/PAS based on psychological suffering | 90 patients had more than one disorder  Depression (N=58) and Personality disorders (N=50) were the most common diagnoses  12 Autism Spectrum Disorder (all Asperger syndrome) : a neglected disease burden  48/100 euthanasia requests were accepted, of which N= 35 were carried out, N= 2 committed suicide before the procedure got implemented, N=11 decided to either postpone or cancel the euthanasia procedure |
| --- | --- | --- | --- |
| Kim et al., 2016 | Netherlands | Review of psychiatric E/PAS case summeries 2011-2014 | Characteristics of the 66 cases reviewed  70% women  32% 70 years or older, 44% (n = 29) 50 to 70 years old, 24% (n = 16) 30 to 50 years old  55% depressive disorders  52% personality disorders  52% previous suicide attempts  41%of physicians performing EAS were psychiatrists, the rest usually general practitioners  89% consultation with another independent psychiatrist |
| Dierickx et al., 2017 | Belgium | Analysis of the anonymous database of euthanasia cases 2002-2013 | 179 reported euthanasia cases with a psychiatric disorder or dementia as the sole diagnosis (0.5% of all cases, increasing to 3.0% in 2013)  Mood disorders (N = 83, 46.4%)  Dementia (N = 62, 34.6%)  Other psychiatric disorders (N = 22, 12.3%)  Mood disorders with another psychiatric disorder (N = 12, 6.7%) |
| Mehlum et al., 2020 | Norway | Narrative review on E/PAS in personality disorders | Granting E/PAS when patients’ suffering perceived as chronic, unbearable and untreatable without prospect of improvement.  People with personality disorders had in very few cases received any relevant evidence-based treatment : improving clinical practice may impact on E/PAS requests and quality of life. |
| Dom et al., 2020 | Belgium, Netherlands | Narrative Review on the Belgian and Dutch E/PAS experiences in the previous decade | Increased prevalence of E/PAS since 2012  Psychiatric E/PAS 1% of all E/PAS cases :  77% female  74% older than 50 years  Most prevalent diagnoses : mood disorders (66%), personality disorders (54%), anxiety disorders (29%), and eating disorders (20%).  More than one psychiatric disorder in 71%  History of suicide attempts in 34%  Supporting E/PAS for psychiatric disorders : general public : 53% ; physicians : 20% (medical specialists), 47% (general practitioners) and 39% (psychiatrists) |
| Calati et al., 2021 | Netherlands, Belgium, Switzerland | Systematic review on 24 papers up to September 2020 | Netherlands :  Patients requesting E/PAS were mostly women (70–77%)  More than one psychiatric disorder in 56–97%  Mood disorders (55–70%) and personality disorders (52–54%) were the most represented.  History of suicide attempts in 34–52%.  At least one comorbid medical condition in 37–62%  Belgium :  Mostly women (75%)  Singole psychiatric disorder in 71% |

**References**

Calati, R., Olié, E., Dassa, D., Gramaglia, C., Guillaume, S., Madeddu, F., Courtet, P., 2021. Euthanasia and assisted suicide in psychiatric patients: A systematic review of the literature. J. Psychiatr. Res. 135, 153–173. https://doi.org/10.1016/j.jpsychires.2020.12.006

Dierickx, S., Deliens, L., Cohen, J., Chambaere, K., 2017. Euthanasia for people with psychiatric disorders or dementia in Belgium: Analysis of officially reported cases. BMC Psychiatry 17, 1–9. https://doi.org/10.1186/s12888-017-1369-0

Dom, G., Stoop, H., Haekens, A., Sterckx, S., 2020. Euthanasia and assisted suicide in the context of psychiatric disorders: Sharing experiences from the Low Countries. Psychiatr. Pol. 54, 661–672. https://doi.org/10.12740/PP/124078

Kim, S.Y.H., De Vries, R.G., Peteet, J.R., 2016. Euthanasia and Assisted Suicide of Patients With Psychiatric Disorders in the Netherlands 2011 to 2014. JAMA Psychiatry 73, 362. https://doi.org/10.1001/jamapsychiatry.2015.2887

Mehlum, L., Schmahl, C., Berens, A., Doering, S., Hutsebaut, J., Kaera, A., Kramer, U., Moran, P.A., Renneberg, B., Ribaudi, J.S., Simonsen, S., Swales, M., Taubner, S., Di Giacomo, E., 2020. Euthanasia and assisted suicide in patients with personality disorders: A review of current practice and challenges. Borderline Personal. Disord. Emot. Dysregulation 7, 1–7. https://doi.org/10.1186/s40479-020-00131-9

Thienpont, L., Verhofstadt, M., Van Loon, T., Distelmans, W., Audenaert, K., De Deyn, P.P., 2015. Euthanasia requests, procedures and outcomes for 100 Belgian patients suffering from psychiatric disorders: A retrospective, descriptive study. BMJ Open 5. https://doi.org/10.1136/bmjopen-2014-007454
